# Burden and Characteristics of Respiratory Syncytial Virus‐Associated Bronchiolitis in Hospitalized Infants in Italy: A Systematic Review

**DOI:** 10.1002/iid3.70420

**Published:** 2026-04-14

**Authors:** Angela Bechini, Cristina Salvati, Marco Del Riccio, Benedetta Bonito, Enrica Stancanelli, Mario Bruschi, Giulia Ionita, Johanna Iamarino, Davide Bentivegna, Primo Buscemi, Giulia Ciardi, Claudia Cosma, Lorenzo Stacchini, Manjola Bega, Annamaria Schirripa, Lorenzo Bertizzolo, Barbara Muzii, Maria Vittoria Azzi, Salvatore Parisi, Francesca Trippi, Paolo Bonanni, Sara Boccalini

**Affiliations:** ^1^ Department of Health Sciences University of Florence Florence Italy; ^2^ Medical Specialization School of Hygiene and Preventive Medicine University of Florence Florence Italy; ^3^ Sanofi Lyon France; ^4^ Sanofi Milan Italy

**Keywords:** respiratory infections, respiratory syncytial virus, RSV genotyping, RSV pediatric burden, RSV prevention, RSV seasonality

## Abstract

**Background:**

Respiratory syncytial virus (RSV) is the main cause of bronchiolitis in infants. This systematic review aimed to evaluate the burden of RSV‐associated bronchiolitis among hospitalized Italian infants between 2000 and 2023.

**Methods:**

A comprehensive literature search identified studies examining RSV‐related hospitalizations for bronchiolitis in children aged 0–59 months. Eligible studies met the following criteria: conducted in Italy, focused on children, reported on RSV prevalence, co‐infections, genotype distribution (RSV‐A, RSV‐B), and seasonal trends, and published in English or Italian. Data extraction focused on study design, infant characteristics (e.g., age, preterm birth), and RSV detection methods.

**Results:**

Twenty‐four studies were included. Infants under 12 months were most affected. RSV was the primary pathogen identified, though co‐infections with other respiratory viruses, such as human rhinovirus, were common. RSV infections typically peaked in late autumn and winter, but the COVID‐19 pandemic altered these patterns. This review highlights the significant burden of RSV‐associated bronchiolitis in Italian infants. While RSV remains the primary pathogen, co‐infections and pandemic related factors have altered its epidemiological trends.

**Conclusions:**

Nationwide RSV immunization programmes and improved diagnostics are crucial to ease the strain on pediatric intensive care units. Recent recommendations for widespread RSV long‐acting monoclonal antibody use in infants offer promising solutions to address this challenge.

## Graphical abstract

This review highlights the significant burden of RSV‐associated bronchiolitis in Italian infants 0‐59 months. While RSV remains the primary pathogen, co‐infections and pandemic‐related factors have altered its epidemiological trends.

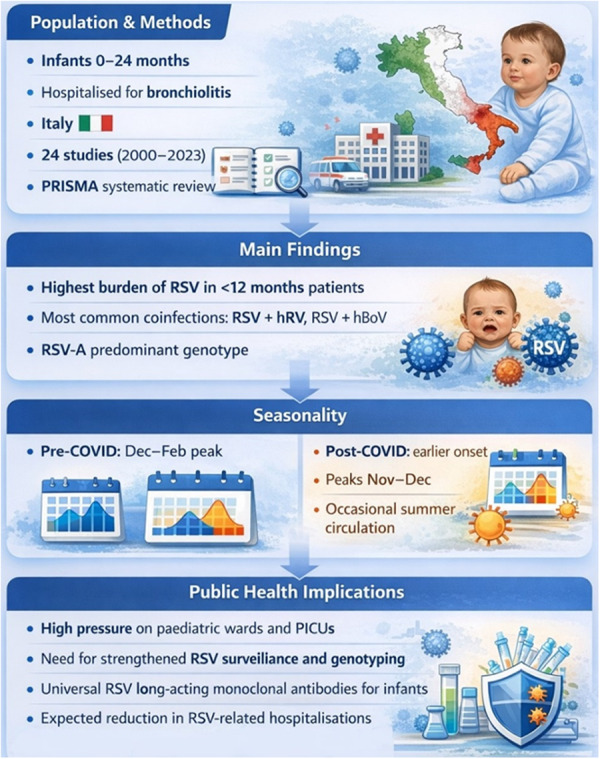

## 1 Introduction

Bronchiolitis is a common respiratory infection that primarily affects infants and young children, typically under 2 years of age, characterized by inflammation and congestion in the small airways (bronchioles) of the lungs [1]. This condition is usually caused by a viral infection, most commonly respiratory syncytial virus (RSV); however, it can also be triggered by other viruses such as rhinovirus or adenovirus [2]. Symptoms of bronchiolitis include wheezing, coughing, difficulty breathing, and a runny nose; while most cases are mild and can be managed at home, severe cases could require hospitalization, especially in very young infants or those with underlying health conditions [3].

Understanding the burden and characteristics of RSV‐associated bronchiolitis in hospitalized infants is essential due to its significant clinical, economic, and public health implications: RSV is the most common viral pathogen responsible for bronchiolitis, which is the leading cause of hospitalization among infants, particularly those under 1 year of age [4, 5]. Moreover, the high prevalence of RSV infections during the winter months leads to seasonal surges in hospital admissions, putting substantial strain on healthcare systems, particularly in pediatric wards and Intensive Care Units (ICU). Hospitalization for RSV‐associated bronchiolitis indicates severe disease and is associated with increased risks of long‐term respiratory complications, including recurrent wheezing and asthma [6, 7]. These potential outcomes highlight the need for early and effective management strategies. Proper interventions can help mitigate short‐term morbidity and long‐term health consequences in affected infants. Furthermore, the economic burden associated with RSV hospitalizations, including direct medical cost, such as hospital stays and treatment, as well as indirect costs (parental work absences), represents a significant concern for families and healthcare systems alike [8].

Moreover, RSV‐associated bronchiolitis represents a paradigmatic model of virus‐induced airway inflammation in early life, with important implications for immune development. RSV infection triggers an intense innate immune response in the immature airway, characterized by epithelial activation, cytokine and chemokine release, and recruitment of neutrophils and lymphocytes, which collectively contribute to airway obstruction and disease severity. Importantly, an imbalanced antiviral immune response, often skewed toward a type 2 inflammatory profile with impaired interferon signaling, has been associated with more severe bronchiolitis and with long‐term respiratory sequelae such as recurrent wheezing and asthma. The high frequency of RSV hospitalizations during the first months of life underscores a critical window in which virus‐driven inflammation may shape immune trajectories and lung health. In this context, defining the epidemiological burden, age distribution, viral subtypes, and coinfections associated with RSV bronchiolitis is highly relevant for immunology and inflammation research, as these factors influence host immune responses and may modulate both acute disease expression and long‐term immunopathological outcomes. [9] (Supporting Information File 7).

In the context of Italy, with the availability of new RSV monoclonal antibodies [10, 11], understanding the specific burden and characteristics of RSV‐associated bronchiolitis is now crucial. As a matter of fact, it is now crucial to have robust, localized epidemiological data to guide immunization strategies and assess the potential impact of these interventions on reducing RSV‐associated burden of disease in children. Considering the above, the aim of this systematic review was to gather evidence on the burden of RSV infection in infants of 0–59 months old, hospitalized with a diagnosis of bronchiolitis, in Italy.

## 2 Materials and Methods

We aimed to identify the proportion of RSV‐infected patients hospitalized with bronchiolitis, the age distribution of these cases, the percentage of positive cases associated with RSV subgroups A or B, the occurrence and most frequent types of co‐infections, and the seasonal pattern of RSV in Italy.

This study adhered to the Preferred Reporting Items for Systematic Reviews and Meta‐Analyzes (PRISMA) guidelines (Supporting Information File 1). The protocol was registered on the PROSPERO register of systematic reviews on August 8, 2021, with the registration number CRD42021248309. The registration details can be found at this link: https://www.crd.york.ac.uk/prospero/display_record.php?RecordID=248309.

The search of online databases included studies from January 1, 2000, until the data extraction was conducted on December 31, 2023. The researchers consulted the following databases: PubMed, Embase, Scopus, and the International HTA Database. The search query was tailored to each database to maximize sensitivity and avoid omitting relevant articles and, for PubMed, it reads as follows: (RSV OR hRSV OR Respiratory Syncytial Virus OR bronchiolitis OR ILI OR ARI OR SARI OR respiratory infection OR “respiratory tract infection” OR RTI OR URI OR URTI OR LRI OR LRTI OR “Viral pneumonia” OR otitis) AND (burden OR impact OR epidemiol* OR economic OR cost* OR hospital* OR incidence OR prevalence OR diagnos* OR diagnosis OR “laboratory confirm*” OR surveillance) AND (Italy OR Italian OR Italians OR Ital*) AND (pediatric OR child* OR toddler* OR newborn* OR infant* OR preterm OR pediatric*).

### 2.1 Inclusion and Exclusion Criteria

The inclusion criteria for the study involved research conducted in Italy, with manuscripts published between January 2000 and December 2023, specifically focusing on the pediatric population aged 0 to 59 months who were hospitalized with a diagnosis of bronchiolitis. Furthermore, all studies had to include laboratory confirmation of RSV infection. Original articles reporting results of observational studies and clinical trials were considered for inclusion, while reviews, letters, editorials, and other records not reporting data were excluded from the final database. Only studies written in English or Italian were included in the final selection. Moreover, studies were excluded when a substantial overlap of the study population (> 80%), based on study period, setting, and inclusion criteria, was identified. In such cases, the most recent study with the largest sample size or longest observation period was retained, in accordance with PRISMA guidelines.

### 2.2 Screening and Study Selection

All extracted studies were compiled in Microsoft Excel (Microsoft 365 MSO, Microsoft Corporation, 2022), and duplicates were removed during this process. Before the eligibility assessment, four reviewers, working in pairs, conducted a double‐blind screening of titles, abstracts, and full texts. Any disagreements that arose were resolved by a fifth investigator. Furthermore, the reference lists of all eligible studies and previously published literature reviews were examined to identify additional articles that covered the same topic.

### 2.3 Data Retrieval and Analysis

After completing the process of selecting records, all articles were thoroughly read and analyzed. Information on study design, which included cohort, case‐control, and cross‐sectional studies, was extracted along with geographical setting and observation timeframe, participant characteristics like age range and sample sizes. To ensure accuracy, data entries underwent cross‐validation by multiple reviewers: the data extraction process also involved identifying the clinical diagnoses associated with hospitalization, analyzing how RSV cases were distributed among various age groups, and documenting available details about RSV subtypes (RSV‐A and RSV‐B). Whenever feasible, data pertaining to concurrent infections with other pathogens and the seasonal trends of RSV infection were also collected. Subsequently, the extracted data were meticulously organized into designated tables, laying the groundwork for subsequent analysis. All studies were classified as prospective and retrospective based on what was reported in the Materials and Methods section for each record. The narrative analysis within this systematic review focused on the key themes identified during the data extraction phase, namely age distribution, distribution of subtypes, and instances of co‐infections among hospitalized cases. The analysis of the studies obtained from the selection process was performed considering the following parameters: RSV cases proportion in children hospitalized for bronchiolitis, coinfections, RSV‐A and RSV‐B and RSV seasonality.

### 2.4 Quality Assessment of the Selected Studies

To assess the methodological quality of the included studies, an adapted Newcastle‐Ottawa Scale (NOS) by combining the scores from the Selection and Comparability categories into a single summed value. Integrating these categories simplified the evaluation process while preserving methodological rigor. Each study was assigned a score within the NOS criteria, with a top score of 9 indicating the least bias risk.

## 3 Results

### 3.1 Selected Studies

A total number of 28,436 records were downloaded from the following databases: PubMed, Embase, Scopus and International HTA database. Supporting Information File 1 shows the flow‐diagram which describes the studies selection process conducted according to PRISMA guidelines (Supporting Information File 2). Duplicates (*n* = 3892) and other records (*n* = 3066) not in Italian or English were removed; 22,478 records were screened by title and abstract. Records then excluded at this stage were 21,731, leading to 747 records.

Following the filtering procedure, a total of 24 articles [12, 13, 14, 15, 16, 17, 18, 19, 20, 21, 22, 23, 24, 25, 26, 27, 28, 29, 30, 31, 32, 33, 34, 35] were included in this research (Supporting Information File 3). Every study examined in this systematic review received a high score rating (7 or 8) during the assessment (Supporting Information File 4). Out of the chosen articles, there were 10 prospective studies and 14 retrospective studies. During the selection process, some studies were excluded because they were comparable to more recent studies of the same research team that included subsequent epidemic seasons. (Supporting Information File 5). As a result, studies with a lower number of children included were removed. Precisely, Petrarca et al. (2021) [22] was selected, while two other studies [36, 37] with overlapping data were excluded. Similarly, Camporesi et al. (2023) [13] was included and another study with comparable data, was excluded [38], as for Midulla et al. (2011) [33], that was included while two other studies were excluded [39, 40]. However, in the case of Scagnolari et al. (2009) and Scagnolari et al. (2012), both studies were included despite a certain overlap, as they have different inclusion criteria and report data of interest [31, 34]. Sensitivity analyses were conducted qualitatively due to heterogeneity in study design and outcome reporting. Across all scenarios, the direction and interpretation of the findings remained consistent, confirming the robustness of the results.

All the selected studies present a population of hospitalized children for bronchiolitis aged between 0 and 2 years with most of them (19 out of 24 studies) under 12 months of age. None of the included studies reported data for children of 3–5 years old, although the search strategy was deliberately broad so as not to omit relevant articles. The population of children included in the different studies shows heterogeneity about the presence of children born at term and comorbidity. For some studies, the percentage of children born preterm and the presence or absence of other pathological conditions is expressed (Supporting Information File 6). In these studies, the percentage of premature children or with comorbidity has always been lower than 20% of the total population [12, 13, 18, 19, 20, 23]. The study of Vittucci et al. reports chronic heart disease and chronic lung disease among the conditions most frequently found in comorbidity [18]. In the other studies, preterm birth or the presence of comorbidity is included among the exclusion criteria or is not detailed.

### 3.2 RSV‐Cases Proportion in Children Hospitalized for Bronchiolitis

**Figure 1:**
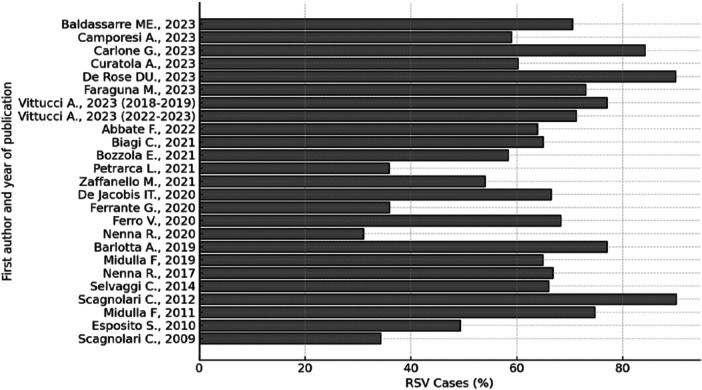
RSV cases (%) in children hospitalized for bronchiolitis. Please note where not otherwise indicated, the percentages of RSV cases refer to all epidemic seasons considered by each individual study and were extracted from each article as reported.

The main findings regarding the proportion of RSV cases (expressed as a percentage, reported by each study) in children hospitalized for bronchiolitis are summarized in Figure 1.

If we consider the time span in which the studies included in this review were conducted, the least recent one [35] is a study performed during the interval of months December‐March from 2006 to 2008. All hospitalized patients with bronchiolitis were < 1 year of age and 54 out of 157 RSV positive with a 34.4% of RSV‐cases proportion [35]. On the other hand, the most recent study [16] concerns a population (< 3 months of age) examined in the period October 2022–March 2023. In this case, RSV rate was 90% (54/60) with a median age at admission of 26 days (range: 18–41 days).

### 3.3 Coinfections in Children Hospitalized for Bronchiolitis

Regarding coinfections, 10 out of 24 studies conducted during 2004–2023, investigated co‐infections (Table 1).

**Table 1:** Coinfections in children hospitalized for bronchiolitis.

First author and year of publication	Coinfections (%, *N*)	Pathogens in coinfection	%, *N*
*Carlone G, 2023*	15.3% (32/209) ‡	RSV + pneumo	4.3% (9/209)
		RSV + hRV	3.3% (7/209)
		RSV + hBoV	1.9% (4/209)
		RSV + SARS‐CoV‐2	1% (2/209)
		RSV + AdV	1% (2/209)
		RSV + PIV	1% (2/209)
		RSV + hRV + pneumo	0.5% (1/209)
		RSV + PIV + pneumo	0.5% (1/209)
		hRV + hBoV	1% (2/209)
		hRV + Coronavirus + hBoV + HiB + any other pathogen †	1% (2/209)
*De Rose D.U., 2023*	30% (18/60)	RSV + pneumo + Hib	1.6% (1/60)
		RSV + Coronavirus 229E + Hib	1.6% (1/60)
		RSV + B Parapertussis	1.6% (1/60)
		Any other viral coinfections †	25% (15/60)
*Abbate F., 2022*	7.7% (24/313)	RSV + hRV	4.5% (14/313)
		RSV + AdV	0.6% (2/313)
		RSV + hBoV	0.6% (2/313)
		RSV + Coronavirus	0.6% (2/313)
		RSV + hMpV	0.3% (1/313)
		hRV + bocavirus	0.3% (1/313)
		hRV + hMpV	0.3% (1/313)
		hRV + coronavirus	0.3% (1/313)
*Bozzola E., 2021*	23.7%(126/531)	Viral coinfections †	not available
*Petrarca L., 2021*	6.3% (83/1312)	RSV + hRV (coinfection most identified)	not available
*Ferro V., 2020*	41.7% (50/120)	RSV + any other virus †	not available
		hRV + any other virus †	not available
*Midulla F., 2019*	not available	RSV + any other pathogen †	13.1% (54/359)
*Nenna R., 2017*	11.1% (39/351)	RSV + hBoV	3.70% (13/351)
		RSV + hRV	3.70% (13/351)
		RSV + hMpV	0.57% (2/351)
		RSV + IVA	0.57% (2/351)
		RSV + PIV type 3	0.28% (1/351)
		RSV + RV + hMpV	0.28% (1/351)
		RV + hMpV	1.14% (4/351)
		RV + PIV type 3	0.28% (1/351)
		hBoV + hMpV	0.28% (1/351)
		hBoV + PIV	0.28% (1/351)
*Midulla F., 2011*	11.5% (20/174)	RSV + hBoV	8.6% (15/174)
		RSV + IV	0.6% (1/174)
		RSV + PIV	0.6% (1/174)
		RSV + hRV	0.6% (1/174)
		hMpV + hBoV	0.6% (1/174)
		hMpV + hRV	0.6% (1/174)
*Scagnolari C., 2009*	5.06% (8/157)	RSV + hBoV	4.45% (7/157)
		RSV + hRV	0.63% (1/157)

Abbreviations: AdV, adenovirus; hBoV, human bocavirus; hMPV, human metapneumovirus; Hib, haemophilus influenzae type B; hRV, human rhinovirus; IV/IVA, influenza virus A; PIV, parainfluenza virus; pneumo, pneumococcus; †, not explicitly stated in the article; ‡, obtained from the data available in the article.

Several studies provide comprehensive data on overall coinfection rates recorded in children with bronchiolitis. For example, Carlone G. et al. [14] report a coinfection rate of 20.6%, while De Rose D.U. et al. [16] observe a higher proportion at 30%. Other studies, such as Bozzola E. et al. [21] and Nenna R. et al. (2017) [30], indicate rates of 23.7% and 11.1%, respectively. When examining specific coinfections, a variety of pathogens have been identified together with RSV. For example, RSV combined with human rhinovirus (hRV) is frequently reported, with rates ranging from 3.7% [30] to 4.5% [19]. Similarly, RSV and human bocavirus (hBoV) coinfections are prominent, with frequencies covering from 1.9% [14] to 8.6% [33]. RSV and human metapneumovirus (hMPV) combinations are less common, reported at 0.3% [19] and 0.57% [30].

### 3.4 RSV Genotyping: RSV‐A and RSV‐B

**Figure 2:**
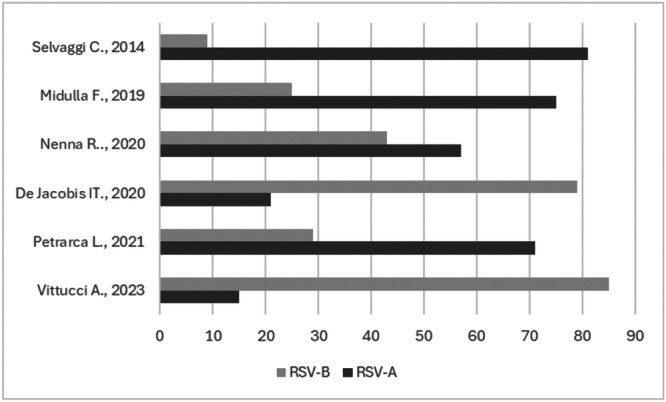
RSV genotypes in children hospitalized for bronchiolitis (%).

Some studies genotypically characterized the RSV type A or B (Figure 2).

In four out of six studies, the majority of cases of RSV infections appear to be due to subgroup A [22, 27, 29, 31] although the study published in 2020 by De Jacobis et al. (2020) carried out during January ‐ December 2017, reported RSV‐B as the primary circulating subtype [24], as also reported in the study by Vittucci et al. [18].

### 3.5 Seasonality of RSV‐Bronchiolitis Cases

Regarding RSV circulation, five out of 24 studies reported its seasonality (Table 2) [12, 13, 17, 18, 30].

**Table 2:** Seasonality of RSV‐bronchiolitis cases.

First author and year of publication	Seasonal trends
*Baldassarre ME., 2023*	RSV bronchiolitis peaked in November 2021 (207 cases; 7.4/1000 children).
*Camporesi A., 2023*	2021–22: cases started in July, peaked Nov–Dec, declined sharply after mid‐Dec.
	2022–23: cases began in Oct, peaked late Dec–early Jan, declined by late Jan.
*Faraguna M., 2023*	2021–22: cases peaked in Nov (57% of season total), showing an anticipated trend compared to prior seasons.
*Vittucci A., 2023*	2022–23: epidemic lasted 17 weeks (wk 42–wk 6, 2023), peaking in the 1st week of 2023.
	2018–19: epidemic spanned 18 weeks (wk 47–wk 12, 2019), peaking in wk 52.
*Nenna R., 2017*	RSV epidemics consistently showed a winter trend, peaking Dec–Feb over 10 years. Significant increases in Jan, Feb, Mar, Apr, and Dec.

Abbreviation: Wk, week.

The distribution of cases recorded in different studies shows a consistent trend of higher cases being reported during the winter months, from November to March. Before 2021, cases peaked in January and February, whereas from 2021 onwards, peaks shifted to November and December. However, during the 2021–2022 season, the first cases were noted in the middle of the summer (July and August), an anomalous report at our latitudes, deviating slightly from the overall pattern, as seen in the case of Camporesi A. et al. [13].

## 4 Discussion

This systematic review aimed to assess the burden of RSV‐associated bronchiolitis in hospitalized infants aged 0–59 months in Italy between 2000 and 2023. Bronchiolitis is the leading cause of hospitalization for respiratory illness in children under 2 years of age, primarily caused by RSV infection, with the highest incidence occurring in infants under 6 months [41].

Among the 24 studies reviewed, infants under 12 months were the most affected by RSV‐associated bronchiolitis. Although the search strategy was intentionally broad to encompass data from patients aged 0–59 months, the findings indicate that the studies on hospital burden for this particular diagnosis in Italy is primarily concentrated within the first 12 months of life. Thus, the results of our review cannot be extended to the 2–5 years age group. This finding aligns with other studies, including a recent European study estimating that 78.3% of RSV‐related hospitalizations in Italy (mostly due to bronchiolitis) occur in this age group [4]. Similarly, a scoping review of studies published between 2009 and 2021 found that most RSV‐related hospitalizations occur in infants under 1 year, with the highest concentration in those aged 0–2 months [42]. The relationship between RSV‐associated hospitalizations and bronchiolitis is closely linked: in fact, when focusing specifically on bronchiolitis, the studies included in this review confirm that hospitalized children with bronchiolitis in Italy are predominantly infected with RSV, which is identified as the primary causative agent in most cases. This trend is also observed internationally, where RSV is estimated to be responsible for 60%–80% of bronchiolitis cases in infants, particularly during peak viral seasons between autumn and spring [43]. However, it is important to recognize that co‐infections with other viruses may also be observed in children with bronchiolitis. In our review, it is possible to observe hRV and hBoV as the most common viruses found in co‐infections with RSV: this confirms findings from previous studies that reported a high co‐detections rate with hRV and hBoV [44, 45] in children admitted to hospital and PICU with RSV related bronchiolitis. Co‐infections are important to recognize because they can exacerbate the severity of bronchiolitis, possibly leading to longer hospital stays, increased need for respiratory support, and higher ICU admission rates [46, 47]. Comprehensive diagnostic approaches are crucial to detect all potential pathogens involved, as overlooking co‐infections may lead to suboptimal treatment and poorer outcomes [48, 49].

As regards the molecular characterization of RSV in pediatric patients hospitalized for bronchiolitis in Italy, the available data are scarce: four out of six studies [22, 27, 29, 31] reported a predominance of genotype A over B, overall, in the investigated populations. In detail, the molecular characterization of RSV‐positive samples revealed a greater presence of the genotype A. As a matter of fact, Midulla et al. 2019 [28] reported that RSV‐A virus dominated in 8 seasons (from 2005 to 2006 to 2016–2017) while RSV‐B and RSV‐A cases were comparable in 3 seasons (2010–2011, 2015–2016, 2016–2017). A recently published systematic review reported that, on a global level, most collected and sequenced samples (from 83 countries) were attributed to RSV‐A infections. The authors also observed a pattern with continuous seasons where genotype A predominated and others with alternating genotypes A and B. The predominant RSV‐A genotype was NA1 (76.3%) while for RSV‐B it was BA (70.6%) [50].

Seasonality of RSV infections demonstrated significant variability across included studies, with most peaks occurring between late autumn and winter, typically from November to January. However, the 2021–2022 season showed atypical patterns, such as bronchiolitis cases appearing as early as July and August, particularly noted by Camporesi et al. [13]. This disruption is known to have been influenced by the COVID‐19 pandemic, which altered the seasonality of many respiratory viruses due to public health measures like lockdowns. Additionally, epidemic lengths varied, with some seasons lasting 17 to 18 weeks, showing a rapid decline in cases post‐peak [18, 51].

This study has a number of limitations. Our data were drawn from investigations with different and various study designs, which may have introduced heterogeneity in the results and limit the ability to make direct comparisons across studies. Although the initial aim of the review was to include children up to 59 months of age, it emerged that none of the 24 selected studies reported data for the 3–5 age group, limiting the evidence collected to the population aged 0–24 months. Therefore, the results of this review cannot be generalized to the upper end of the expected age range (> 24 months), as the RSV burden in this specific pediatric subgroup remains unassessed by the Italian literature focused on hospitalized bronchiolitis.

The broad temporal range of the data, spanning from 2000 to 2023, is useful to have a deep comprehension of RSV‐associated burden in Italy over two decades, but may further complicates interpretation, as changes in diagnostic methods and public health policies. While formal temporal subgroup analyses were constrained by heterogeneity and reporting differences, descriptive assessment indicates a trend toward higher RSV detection in more recent studies, likely reflecting the transition from antigen‐based to molecular diagnostics. These temporal variations should be considered when interpreting pooled estimates, and highlight the need for ongoing surveillance and standardized reporting to accurately monitor RSV burden over time. Moreover, external factors, such as the COVID‐19 pandemic, could have influenced the seasonality and prevalence of RSV infection in children hospitalized for bronchiolitis. Furthermore, the molecular characterization of RSV genotypes and RSV seasonality is only partially captured in this systematic review, with just six [18, 22, 24, 27, 29, 31] and five articles [12, 13, 17, 18, 30], respectively, providing data related to these aspects. This review highlighted how data on the genotypes and seasonality of RSV bronchiolitis in hospitalized infants remain limited, particularly for Italy. This gap in knowledge presents a challenge for public health, considering that robust data are essential to developing and refining prevention strategies. With the availability of new preventive tools such as long‐acting monoclonal antibodies (mAbs), recently licensed and approved for use [11], ongoing surveillance is indeed critical to ensure these interventions are used effectively. However, their success will depend on well‐informed public health strategies that are tailored to the epidemiological patterns of RSV.

In the context of Italy, bronchiolitis remains a significant healthcare challenge, particularly during the winter months when RSV and other respiratory viruses circulate heavily. Italian hospitals face seasonal surges in bronchiolitis admissions, which place considerable strain on pediatric intensive care units. Efforts to introduce nationwide RSV immunization programs for infants with RSV long‐acting monoclonal antibody and improve diagnostic capacities are increasingly needed to reduce the burden on the healthcare system [8, 52]. Recently, the Board for Lifecourse Vaccination recommended the widespread use of RSV long‐acting monoclonal antibody for all newborns in Italy [53]. In addition, since the 2024/2025 RSV season, all regions of Italy have adopted a preventive strategy using RSV long‐acting monoclonal antibody to protect all infants, supported by a dedicated fund provided by the Ministry of Health [54]. This preventive measure is expected to reduce the severity and incidence of RSV‐related bronchiolitis, ensuring that infants receive protection during their most vulnerable months, regardless of their region of residence. This measure represents significant progress in addressing a longstanding unmet medical need in Italy, where bronchiolitis hospitalizations due to RSV account for a substantial portion of pediatric admissions during the cold season.

This systematic review adds to the existing literature by providing a comprehensive and Italy‐specific synthesis of RSV‐associated bronchiolitis in hospitalized infants over a long observation period (2000–2023). While previous reviews have primarily focused on the overall burden of RSV hospitalizations or broader pediatric populations, this review uniquely integrates multiple dimensions of RSV epidemiology, including RSV prevalence among hospitalized bronchiolitis cases, patterns of viral co‐infections, distribution of RSV‐A and RSV‐B genotypes, and seasonality trends. Importantly, it also captures the substantial epidemiological changes observed following the COVID‐19 pandemic, which have not been fully addressed in earlier Italian reviews. By consolidating fragmented evidence into a single national overview and interpreting findings in light of recently introduced RSV preventive strategies, this review provides added value for public health planning, surveillance optimization, and the implementation of RSV immunization policies in Italy.

Finally, to address current gaps and support future health policies, a focused research agenda could be proposed. Systematic RSV‐A and RSV‐B genotyping should be integrated into national surveillance networks (e.g., RespiVirNet) to monitor viral evolution following the introduction of long‐acting monoclonal antibodies. In parallel, multi‐season prospective studies are needed to better characterize post‐pandemic changes in RSV epidemiology and seasonality. These efforts are essential to ensure that prevention strategies remain aligned with the evolving epidemiological patterns at the national level.

## Author Contributions

**Conceptualization:** Sara Boccalini, Benedetta Bonito, Marco Del Riccio, Cristina Salvati, Lorenzo Bertizzolo, Salvatore Parisi, Francesca Trippi, Barbara Muzii, Maria Vittoria Azzi, Paolo Bonanni, Angela Bechini. **Search strategy:** Sara Boccalini, Benedetta Bonito, Marco Del Riccio, Lorenzo Bertizzolo, Salvatore Parisi, Francesca Trippi, Barbara Muzii, Maria Vittoria Azzi, Paolo Bonanni, Angela Bechini. **Search literature:** Benedetta Bonito, Cristina Salvati, Enrica Stancanelli, Mario Brusch, Giulia Ionita, Johanna Iamarino, Davide Bentivegna, Paolo Bonanni, Giulia Ciardi, Claudia Cosma, Lorenzo Stacchini, Claudia Cosma, M.C., Annamaria Schirripa, Salvatore Parisi. **Data extraction:** Benedetta Bonito, Cristina Salvati, Enrica Stancanelli, Mario Brusch, Giulia Ionita, Johanna Iamarino, Davide Bentivegna, Paolo Bonanni, Giulia Ciardi, Claudia Cosma, Lorenzo Stacchini, Annamaria Schirripa. **Quality assessment:** Marco Del Riccio, Cristina Salvati, Angela Bechini, Sara Boccalini. **Data analysis:** Benedetta Bonito, Cristina Salvati, Marco Del Riccio, Angela Bechini, Sara Boccalini. **Writing—original draft:** Cristina Salvati, Benedetta Bonito, Marco Del Riccio, Angela Bechini, Sara Boccalini. **Writing—review and editing:** Sara Boccalini, Benedetta Bonito, Cristina Salvati, Enrica Stancanelli, Mario Brusch, Giulia Ionita, Johanna Iamarino, Davide Bentivegna, Paolo Bonanni, Giulia Ciardi, Lorenzo Stacchini, Claudia Cosma, Annamaria Schirripa, Salvatore Parisi, Marco Del Riccio, Lorenzo Bertizzolo, Salvatore Parisi, Barbara Muzii, Maria Vittoria Azzi, Francesca Trippi, Paolo Bonanni, Angela Bechini. All authors have read and agreed to the published version of the article.

## Conflicts of Interest

L.B., S.P., B.M., M.A., and F.P. are employees of Sanofi and may hold shares. The remaining authors declare that the research was conducted in the absence of any commercial or financial relationships that could be construed as a potential conflict of interest.

## Supporting information


**Supporting File 1:** PRISMA checklist.

**Supporting file 2:** PRISMA flow diagram.

**Supporting file 3:** Characteristics of the selected studies.

**Supporting File 4:** NOS.

**Supporting file 5:** The table of excluded studies

**Supporting file 6:** Reporting of preterm births and comorbidities in studies included in the SLR

**Supporting file 7:** Immunization strategies.

## Data Availability

The data that support the findings of this study are available from the corresponding author upon reasonable request.
