# Supplementary material for: Burden and Characteristics of Respiratory Syncytial Virus‐Associated Bronchiolitis in Hospitalized Infants in Italy: A Systematic Review
Source: Immun Inflamm Dis. 2026 Apr 14;14(4):e70420. doi: 10.1002/iid3.70420 (PMC13079949; doi:10.1002/iid3.70420)
Supplement: Supplementary file 2 — Supporting file 2: PRISMA flow diagram. [file IID3-14-e70420-s005.docx]

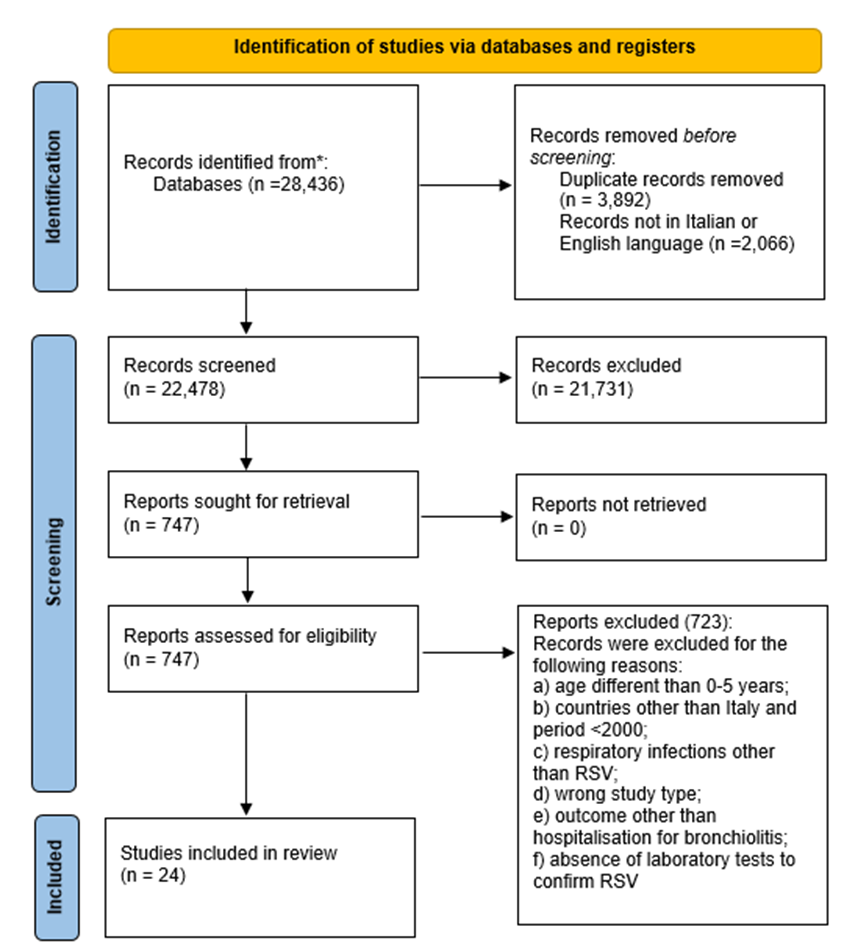


* Databases: Pubmed, Embase, Scopus, International HTA Database

**Supplementary file 2.** Flow diagram for the systematic review (PRISMA statement)
