# Supplementary material for: Burden and Characteristics of Respiratory Syncytial Virus‐Associated Bronchiolitis in Hospitalized Infants in Italy: A Systematic Review
Source: Immun Inflamm Dis. 2026 Apr 14;14(4):e70420. doi: 10.1002/iid3.70420 (PMC13079949; doi:10.1002/iid3.70420)
Supplement: Supplementary file 3 — Supporting file 3: Characteristics of the selected studies. [file IID3-14-e70420-s006.docx]

**Supplementary file 3.** Characteristics of the selected studies

| **First author and year of publication** | **Study centre** | **Study design** | **Time of observation^a^** | **Age (months or days)** | **Total sample size (N) ^b^** |
| --- | --- | --- | --- | --- | --- |
| *Baldassarre ME., 2023* | Apulia region | retrospective | January-December 2021 | 0-12 m | 349 |
| *Camporesi A., 2023* | Children’s Hospital “Vittore Buzzi” (Milan); A. Gemelli hospital (Rome) | prospective | July 2021-31 March 2022; July 2022-March 2023 | <24 m | 900* |
| *Carlone G., 2023* | S. Spirito Hospital,  (Pescara); Children’s Hospital Giovanni  XXIII, (Bari); AUSL Romagna, (Forlì); University of Chieti (Chieti) | retrospective | October 2021- February 2022 | ≤24 m | 214* |
| *Curatola A., 2023* | A. Gemelli Hospital (Rome) | retrospective | September 2021- March 2022; and same months in 2020-2021, 2018-2019, 2019-2020 | ≤ 24m | 937* |
| *De Rose DU., 2023* | “Bambino Gesù” Children’s Hospital IRCCS, (Rome) | retrospective | October 2022-March 2023 | <3 m | 60 |
| *Faraguna M., 2023* | San Gerardo Hospital Fondazione (Monza) | retrospective | September to April (2017-2018, 2018-2019, 2019-2020, 2020-2021, 2021-2022) | 0-12 m | 141* |
| *Vittucci A., 2023* | “Bambino Gesù” Children’s Hospital IRCCS (Rome) | retrospective | October-February of the season 2022–2023 and of the 2018–2019 | <12 m | 300 in 2018-2019; 294 in 2022-2023 |
| *Abbate F., 2022* | Pediatrics Unit of the University Hospital of Pisa | retrospective | January 2010-December 2019 | ≤12 m | 346* |
| *Biagi C., 2021* | Pediatric Emergency Unit (PED) of S. Orsola University Hospital (Bologna) | retrospective | October 2010-April 2020 | <12 m | 1249* |
| *Bozzola E., 2021* | “Bambino Gesù” Children’s Hospital IRCCS (Rome) | retrospective | January 2017 - December 2017 | 1- 12m | 531 |
| *Petrarca L., 2021* | Department Maternal Infantile and Urological Sciences, “Sapienza”University of Rome (Rome) | prospective | October-May from 2004 to 2019 | <12m | 1312* |
| *Zaffanello M., 2021* | Pediatrics and Gynecology, University of Verona | retrospective | January 2014 -November 2016 | <24 m | 63* |
| *De Jacobis IT., 2020* | “Bambino Gesù” Children’s Hospital IRCCS (Rome) | retrospective | January - December 2017 | ≤12 m | 388 |
| *Ferrante G., 2020* | “Giovanni Di Cristina” Pediatric Hospital of Palermo | retrospective | November 2012-May 2019 | ≤24m | 401* |
| *Ferro V., 2020* | “Bambino Gesù” Children’s Hospital IRCCS (Rome) | retrospective | September 2016- April 2018 | <12m | 120* |
| *Nenna R., 2020* | Paediatric Department, “Sapienza” University of Rome | prospective | November 2016 -April 2017 and October 2017-April 2018 | <12m | 290* |
| *Barlotta A., 2019* | University Hospital of Padova | prospective | December 2013 - March 2014 | <12m | 41 |
| *Midulla F, 2019* | Pediatric  Emergency Department, Sapienza University of Rome | prospective | From 2005–2006 to 2016–2017 (September–May) | <12m | 998* |
| *Nenna R., 2017* | Pediatric Emergency  Department, “Sapienza” University of Rome | prospective | October- May 2004 - 2014 | <12 m | 723* |
| *Selvaggi C., 2014* | Paediatric Department of Policlinico Umberto I Hospital (Rome) | retrospective | epidemic seasons from 2008 to 2011** | ≤12 m | 118* |
| *Scagnolari C., 2012* | Pediatric Department of Policlinico Umberto I Hospital (Rome) | retrospective | December-March 2006-2010 | Median 2.20 m | 132* |
| *Midulla F, 2011* | Pediatric Emergency  Department, “Sapienza” University of Rome | prospective | October - May (from 2004 to 2009) | <12m; range 7 d–11 m, median age 2 months (cases) | 313* |
| *Esposito S., 2010* | Department of Maternal and Pediatric Sciences of the University of Milan | prospective | December-March 2007-2008, 2008-2009 | 1-12 m | 69* |
| *Scagnolari C., 2009* | Pediatric Department of Policlinico Umberto I Hospital (Rome) | retrospective | December-March 2006-2007, 2007-2008 | mean and median [SD] age, 65  and 46 [62] days | 157* |

*total patients enrolled in all epidemic seasons; ** months were not reported; a) the term “time of observation” pertains to the years during which the children were admitted to the hospital and, more broadly, to the timeframe to which the gathered data pertains; b) total sample size number refers to infants hospitalised for bronchiolitis and does not always equate to the total number of samples actually tested.
