# Supplementary material for: Burden and Characteristics of Respiratory Syncytial Virus‐Associated Bronchiolitis in Hospitalized Infants in Italy: A Systematic Review
Source: Immun Inflamm Dis. 2026 Apr 14;14(4):e70420. doi: 10.1002/iid3.70420 (PMC13079949; doi:10.1002/iid3.70420)
Supplement: Supplementary file 4 — Supporting File 4: NOS. [file IID3-14-e70420-s001.docx]

**Supplementary file 4.** The Newcastle-Ottawa Scale (NOS) for assessing the quality of the selected studies

| **First author and year of publication** | **Selection/ comparability** | **Exposure/outcome** | **Total quality score** |
| --- | --- | --- | --- |
| *Baldassarre ME., 2023* | 5 | 3 | 8 |
| *Camporesi A., 2023* | 5 | 3 | 8 |
| *Carlone G., 2023* | 5 | 3 | 8 |
| *Curatola A., 2023* | 4 | 3 | 7 |
| *De Rose DU., 2023* | 5 | 3 | 8 |
| *Faraguna M., 2023* | 5 | 3 | 8 |
| *Vittucci A., 2023* | 5 | 3 | 8 |
| *Abbate F., 2022* | 5 | 3 | 8 |
| *Biagi C., 2021* | 4 | 3 | 7 |
| *Bozzola E., 2021* | 5 | 3 | 8 |
| *Petrarca L., 2021* | 5 | 3 | 8 |
| *Zaffanello M., 2021* | 4 | 3 | 7 |
| *De Jacobis IT., 2020* | 4 | 3 | 7 |
| *Ferrante G., 2020* | 4 | 3 | 7 |
| *Ferro V., 2020* | 5 | 3 | 8 |
| *Nenna R., 2020* | 5 | 3 | 8 |
| *Barlotta A., 2019* | 4 | 3 | 7 |
| *Midulla F, 2019* | 5 | 3 | 8 |
| *Nenna R., 2017* | 5 | 3 | 8 |
| *Selvaggi C., 2014* | 5 | 3 | 8 |
| *Scagnolari C., 2012* | 4 | 3 | 7 |
| *Midulla F, 2011* | 5 | 3 | 8 |
| *Esposito S., 2010* | 4 | 3 | 7 |
| *Scagnolari C., 2009* | 5 | 3 | 8 |
