# Supplementary material for: Burden and Characteristics of Respiratory Syncytial Virus‐Associated Bronchiolitis in Hospitalized Infants in Italy: A Systematic Review
Source: Immun Inflamm Dis. 2026 Apr 14;14(4):e70420. doi: 10.1002/iid3.70420 (PMC13079949; doi:10.1002/iid3.70420)
Supplement: Supplementary file 5 — Supporting file 5: The table of excluded studies [file IID3-14-e70420-s007.docx]

**Supplementary File 5.** Table of excluded studies

| **Excluded study**  **(first author, year)** | **Included study**  **(first author, year)** | **Estimated overlap** | **Reason for exclusion** |
| --- | --- | --- | --- |
| Petrarca L., 2018 | Petrarca L., 2021 | >80% | Overlapping population from the same research group; shorter observation period and smaller sample size |
| Petrarca L., 2022 | Petrarca L., 2021 | >80% | Substantial overlap of patients; data largely included in the longer and more comprehensive study |
| Camporesi A., 2022 | Camporesi A., 2023 | >80% | Overlapping cohort; later study included additional epidemic seasons and larger sample |
| Midulla F., 2010 | Midulla F., 2011 | >80% | Same clinical setting and population; smaller sample size |
| Midulla F., 2012 | Midulla F., 2011 | >80% | Overlapping study population with similar inclusion criteria |

Studies were excluded when a substantial overlap of the study population (>80%), based on study period, setting, and inclusion criteria, was identified. In such cases, the most recent study with the largest sample size or longest observation period was retained, in accordance with PRISMA recommendations.
