# Supplementary material for: Burden and Characteristics of Respiratory Syncytial Virus‐Associated Bronchiolitis in Hospitalized Infants in Italy: A Systematic Review
Source: Immun Inflamm Dis. 2026 Apr 14;14(4):e70420. doi: 10.1002/iid3.70420 (PMC13079949; doi:10.1002/iid3.70420)
Supplement: Supplementary file 6 — Supporting file 6: Reporting of preterm births and comorbidities in studies included in the SLR [file IID3-14-e70420-s003.docx]

**Supplementary file 6**. Reporting of preterm birth and comorbidities in studies included in the systematic literature review

| **First author (year)** | **Study design** | **Preterm birth reported** | **Definition of preterm birth** | **Preterm prevalence** | **Comorbidities reported** | **Type of comorbidities** | **Comorbidity prevalence** | **Notes** |
| --- | --- | --- | --- | --- | --- | --- | --- | --- |
| Baldassarre (2023) | Retrospective | Yes | GA <37 weeks | <20% | Yes | Chronic conditions (not specified) | <20% | Reported as baseline characteristics |
| Camporesi (2023) | Prospective | Yes | GA <37 weeks | Not specified | Yes | Chronic diseases | Not specified | Limited quantitative detail |
| Carlone (2023) | Retrospective | No | – | – | No | – | – | Not reported |
| Curatola (2023) | Retrospective | No | – | – | No | – | – | Not reported |
| De Rose (2023) | Prospective | No | – | – | No | – | – | Exclusion criteria applied |
| Faraguna (2023) | Retrospective | No | – | – | No | – | – | Not detailed |
| Vittucci (2023) | Prospective | Yes | GA <37 weeks | <20% | Yes | CHD, CLD | <20% | Most detailed comorbidity reporting |
| Abbate (2022) | Retrospective | Yes | GA <37 weeks | <20% | Yes | Chronic conditions | <20% | Baseline data |
| Biagi (2021) | Retrospective | No | – | – | No | – | – | Not reported |
| Bozzola (2021) | Retrospective | No | – | – | No | – | – | Not reported |
| Petrarca (2021) | Prospective | Yes | GA <37 weeks | <20% | Yes | Chronic diseases | <20% | Included in baseline |
| Zaffanello (2021) | Case–control | Yes | GA <37 weeks | Reported | Yes | Perinatal risk factors | Reported | Focus on risk factors |
| De Jacobis (2020) | Prospective | No | – | – | No | – | – | Not reported |
| Ferrante (2020) | Retrospective | No | – | – | No | – | – | Not detailed |
| Ferro (2020) | Prospective | No | – | – | No | – | – | Excluded chronic disease |
| Nenna (2020) | Prospective | No | – | – | No | – | – | Not reported |
| Barlotta (2019) | Prospective | No | – | – | No | – | – | Not reported |
| Midulla (2019) | Prospective | No | – | – | No | – | – | Focus on genotypes |
| Nenna (2017) | Prospective | No | – | – | No | – | – | Not reported |
| Selvaggi (2014) | Prospective | No | – | – | No | – | – | Not detailed |
| Scagnolari (2012) | Prospective | No | – | – | No | – | – | Not reported |
| Scagnolari (2009) | Prospective | No | – | – | No | – | – | Not reported |
| Midulla (2011) | Prospective | No | – | – | No | – | – | Excluded comorbidities |
| Esposito (2010) | Prospective | No | – | – | No | – | – | Excluded chronic disease |

**Abbreviations:** GA, gestational age; CHD, congenital heart disease; CLD, chronic lung disease.
