# Supplementary material for: Burden and Characteristics of Respiratory Syncytial Virus‐Associated Bronchiolitis in Hospitalized Infants in Italy: A Systematic Review
Source: Immun Inflamm Dis. 2026 Apr 14;14(4):e70420. doi: 10.1002/iid3.70420 (PMC13079949; doi:10.1002/iid3.70420)
Supplement: Supplementary file 7 — Supporting file 7: Immunization strategies. [file IID3-14-e70420-s002.docx]

**Supplementary file 7.** Immunization strategies

Immunization strategies, including monoclonal antibodies and vaccines can interrupt the pathogenic cascade. By preventing viral entry or modulating the immune response, these interventions reduce epithelial injury, limit the recruitment of inflammatory cells, and ultimately decrease clinical severity.

Overall, this diagram serves as a conceptual bridge between clinical outcomes and mechanistic understanding, emphasizing potential targets for therapeutic and preventive strategies. This figure strengthens the review’s conceptual framework by visually linking the epidemiological burden to the underlying immune and inflammatory mechanisms.
